# Membranous Nephropathy Associated With an Over-the-Counter Supplement

**DOI:** 10.7759/cureus.110051

**Published:** 2026-06-01

**Authors:** Nalen Naicker, Mohanlal Mohanlal, Mario Madruga, Steve Carlan

**Affiliations:** 1 Internal Medicine, Orlando Regional Medical Center, Orlando, USA; 2 Nephrology, Orlando Regional Medical Center, Orlando, USA; 3 Obstetrics, Orlando Regional Medical Center, Orlando, USA

**Keywords:** alpha lipoic acid, over-the-counter supplement, percutaneous renal biopsy, protein/creatinine, secondary membranous nephropathy

## Abstract

Membranous nephropathy (MN) is a frequent cause of nephrotic syndrome that can result in ongoing proteinuria, elevated serum creatinine, and declining kidney function. Neural epidermal growth factor-like 1 (NELL1) is a target antigen in MN. Alpha-lipoic acid (ALA) is an over-the-counter antioxidant frequently used for neuropathic pain and may be a potential trigger for NELL1-associated MN (NELL1-MN).

A 56-year-old female was seen for recent onset of acute lower extremity swelling and frothy urine. Proteinuria was identified on urinalysis. Laboratory evaluation showed an abnormally high urine protein/creatinine ratio with a normal serum creatinine. Renal biopsy confirmed NELL1-MN. The patient reported taking a dietary supplement containing 600 mg of ALA daily for neuropathy over the preceding three months. The ALA supplement was discontinued, and the patient was monitored closely with labs. At the five-month follow-up, the patient noted improvement in edema, and repeat laboratory evaluations demonstrated complete resolution of proteinuria.

This case suggests that ALA-associated NELL1-MN may be reversible. Once ALA was identified and discontinued, significant improvement occurred without the need for immunosuppression.

## Introduction

Membranous nephropathy (MN) is a frequent cause of nephrotic syndrome, accounting for up to 30% of cases of nephrotic syndrome [1]. In adults, 20 to 25% of MN cases are linked to a variety of conditions, including infections, autoimmune diseases, malignancies, and the use of specific drugs such as nonsteroidal anti-inflammatory drugs (NSAIDs), alpha-lipoic acid (ALA), and certain traditional medicines [2,3].

Neural epidermal growth factor-like 1 (NELL1) is the second most common target antigen in primary MN [2]. If not properly managed, MN can result in ongoing proteinuria, rising serum creatinine, and progressive kidney disease.

The underlying pathogenesis of MN involves the development of autoantibodies targeting either internal or external antigens, leading to damage of the glomerular basement membrane [4]. Most patients with MN (approximately 80%) present with either nephrotic syndrome or asymptomatic proteinuria [5]. To establish a diagnosis of MN, historically, a renal biopsy was required. However, due to the identification of target antigens and the specificity of serologic assays and biomarkers some clinicians are now electing to begin with a serologic-based diagnostic approach [6]. Treatment for MN depends on the patient's risk of disease progression [7].

ALA, an over-the-counter antioxidant commonly found in agents utilized for neuropathic pain, has emerged as a possible precipitating agent for NELL1-associated membranous nephropathy (NELL1-MN) [3]. Evidence suggests a correlation between ALA exposure and the onset of NELL1-MN, with a limited number of biopsy-confirmed cases reported globally in the literature [3,8,9]. Notably, this subset of MN may achieve complete or partial remission without immunosuppressive therapy if the causative factor is promptly identified and stopped.

## Case presentation

A 56-year-old female with a past medical history of hypothyroidism managed with levothyroxine (100 micrograms daily by mouth), neuropathy, and depression treated with sertraline (50 milligrams daily by mouth), was referred to the nephrology clinic for assessment of proteinuria identified on urinalysis. She was not taking estrogen replacement. She had been on gabapentin 300 mg three times daily for three years for her neuropathy. Her symptoms included lower extremity paraesthesia with numbness and tingling, and she had moderate relief. She reported occasional alcohol consumption and denied tobacco or illicit drug use. Her family history was negative for autoimmune diseases or malignancies. Review of systems was notable for recent onset of lower extremity edema and increased foaminess of urine. She denied dyspnea, orthopnea, chest pain, or headaches. She noted increased tingling, or a "pins and needles" sensation, in her lower extremities over a three-month period. It was not serious enough for her to visit a healthcare provider. In addition, she noted a slow progression of leg edema for approximately three weeks before visiting her local primary health provider.

**Figure 1:**
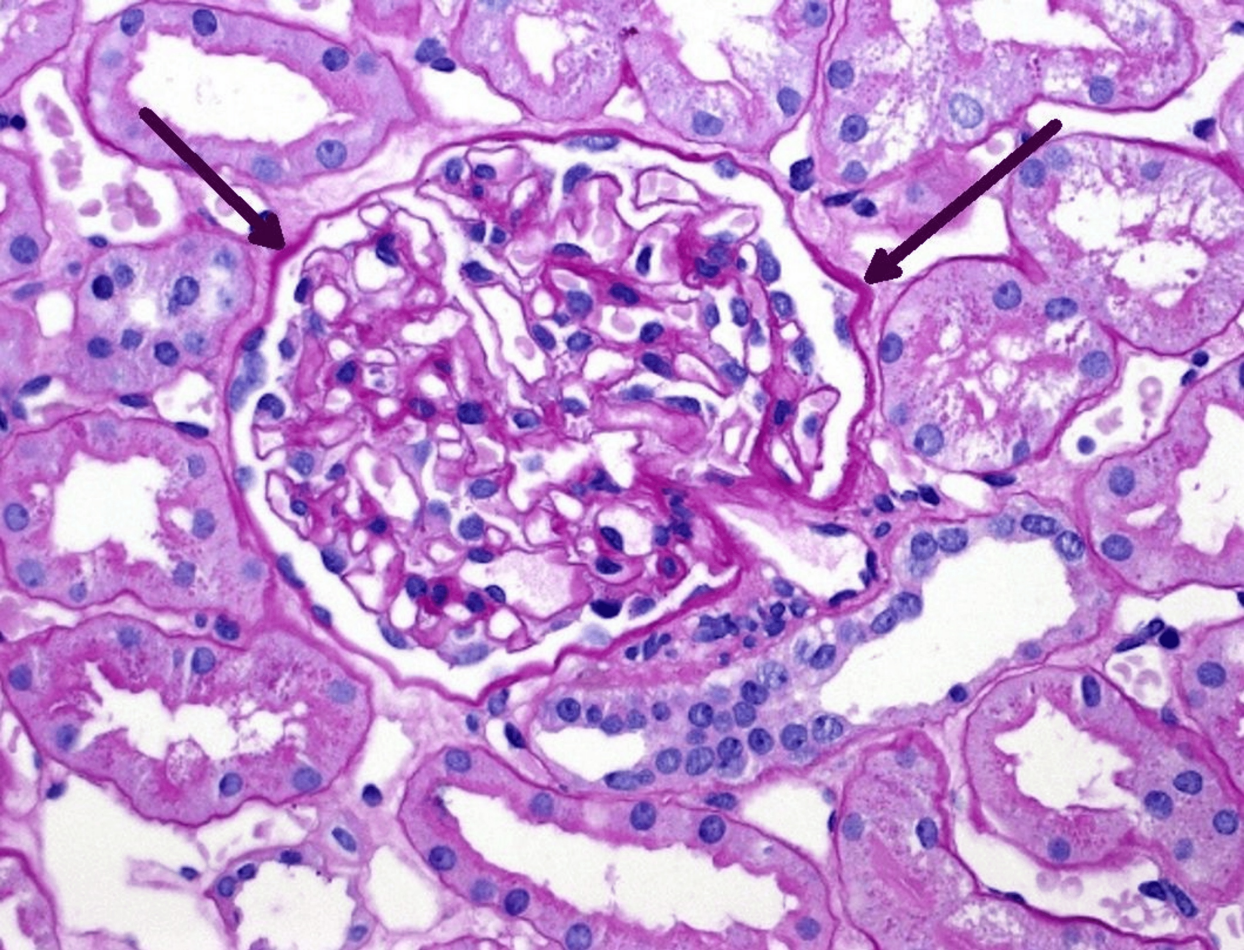
Light microscopy section of glomerulus on Periodic Acid-Schiff stain strongly positive for NELL1 along the glomerular basement membranes (black arrows).

**Figure 2:**
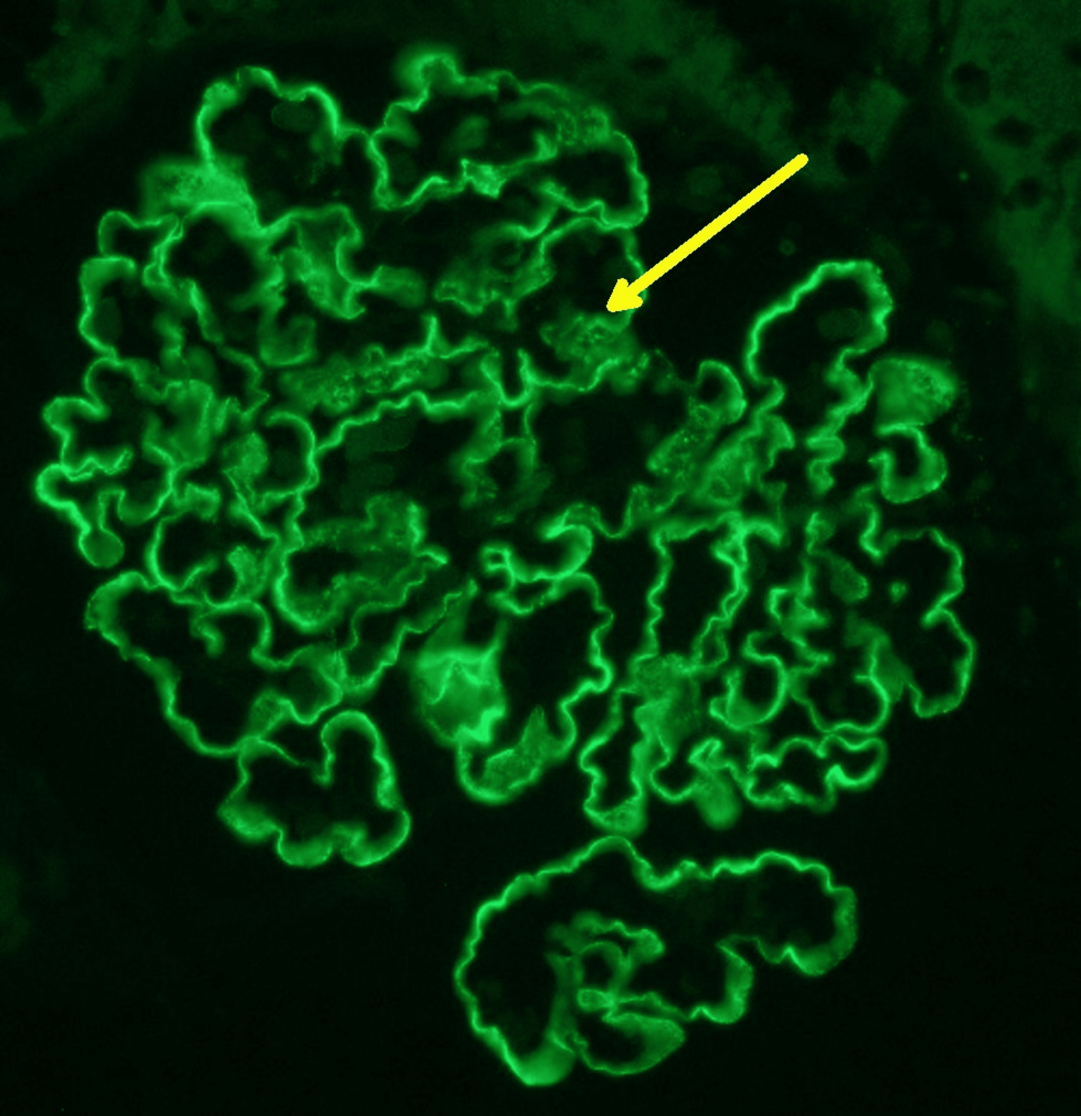
Immunofluorescence of glomeruli revealed diffuse segmental to global granular capillary loop and mesangial staining (yellow arrow) positive for NELL1.

The physical examination demonstrated bilateral pitting edema of the lower extremities. Laboratory evaluation showed a urine protein/creatinine ratio of 5.7 g/g (grams per gram) (normal range < 200 mg/g), and a serum creatinine level of 0.74 mg/dL (milligrams per deciliter) (normal range 0.6 to 1.1 mg/dL). Age-appropriate malignancy screening, including mammography, was negative. CT imaging of the chest, abdomen, and pelvis revealed bilateral small adrenal adenomas; all other findings were unremarkable. Renal biopsy confirmed NELL1-MN (Figure 1, Figure 2).

**Figure 3:**
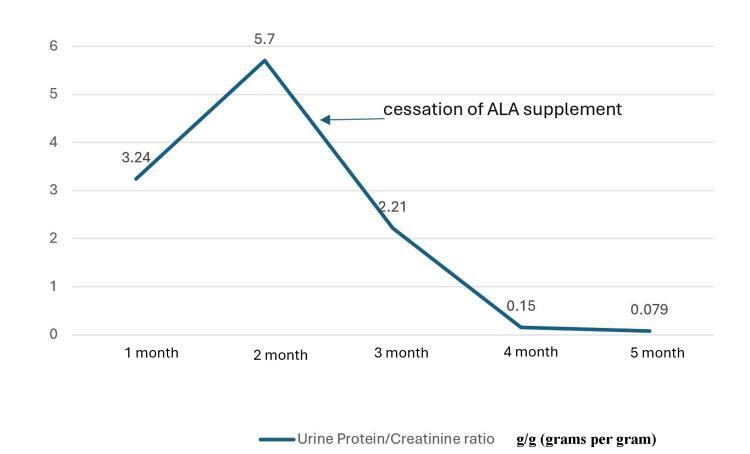
Serial lab values over time showing urine protein/creatinine ratio. ALA: alpha-lipoic acid

Upon additional history, the patient reported taking a dietary supplement containing 600 mg of ALA daily for neuropathy over the preceding three months. She denied the use of any other supplements. Immunosuppressive therapy was not commenced; instead, discontinuation of ALA supplementation was recommended, with close monitoring. At the five-month follow-up, the patient noted improvement in edema, and repeat laboratory evaluations demonstrated complete resolution of proteinuria (Figure 3).

## Discussion

Once NELL1-MN is diagnosed, further investigation is primarily focused on excluding malignancy as the most frequent underlying cause. Nonetheless, NELL1-MN is associated with several factors, including older adults (mean age mid-60s), comorbid conditions such as diabetes mellitus, autoimmune disorders, drug exposures, and isolated cases of supplement use related to ALA [10,11]. By comparison, the patient presented was younger than the typically reported age, had no history of malignancy, and their prescribed medications for antidepressant and hypothyroidism management (sertraline and levothyroxine) have not been documented as causative agents for NELL1-MN in existing literature.

Immunohistochemical staining is utilized to classify the subtype of MN. NELL1 represents the second most prevalent autoantigen in MN, following phospholipase A2 receptor (PLA2R) [12]. In this case, the patient’s kidney biopsy was stained for NELL1, PLA2R, and thrombospondin type 1 domain-containing 7A (THSD7A). The results indicated strong positive staining for NELL1 along the glomerular basement membrane, whereas all other stains were negative. Histologically, NELL1-MN is defined by granular immune complex deposition along glomerular capillary loops, with positive NELL1 staining observed on paraffin immunofluorescence. 

A multi-institutional case series described five patients with ALA-associated NELL1-MN (including four biopsy-confirmed cases), all of whom showed improvement following cessation of ALA, with four achieving complete and one partial remission without immunosuppressive therapy [8]. Additionally, another biopsy-confirmed instance of NELL1-MN was documented in a kidney transplant recipient exposed to both ALA and dimercaptopropane sulfonate, with partial remission observed after withdrawal of both agents [9].

The relationship between ALA use and NELL1-MN development is unclear with respect to both direct causation and dose dependence. Cases involve intake levels from 600 mg/day to 1200 mg/day linked to NELL1-MN [3]. In this instance, our patient disclosed consuming up to 600 mg/day.

The immunologic mechanism by which ALA may cause NELL1-MN is not fully understood. It is thought that ALA’s thiol component may prompt autoimmunity by modifying or exposing the NELL1 antigen on podocytes, leading to NELL1 antibodies forming along the glomerular basement membrane. If this occurs, it may activate complement, leading to podocyte injury and subsequent proteinuria [8].

Supplements are available over the counter and represent a multi-billion-dollar industry regulated under the Dietary Supplement Health and Education Act (DSHEA). Unlike pharmaceutical agents, supplements are not subject to premarket safety and efficacy evaluation by the U.S. Food and Drug Administration (FDA) [13].

A limitation of this report is the inability to establish causality beyond temporal association and observed clinical improvement following ALA withdrawal. Nevertheless, the consistency of this pattern across multiple independent cases reinforces the plausibility of the suspected association.

## Conclusions

In our patient, once ALA was identified and discontinued, significant improvement occurred without the need for immunosuppression. Whether this is a coincidence is not established by one case report. Considering the common use of ALA as a dietary supplement, healthcare providers should regularly ask patients with MN about over-the-counter products. This case adds to the growing body of literature suggesting an association between ALA exposure and NELL1-MN.
